# Transient Silencing of Antibiotic Resistance by Mutation Represents a Significant Potential Source of Unanticipated Therapeutic Failure

**DOI:** 10.1128/mBio.01755-19

**Published:** 2019-10-29

**Authors:** Louise Kime, Christopher P. Randall, Frank I. Banda, Francesc Coll, John Wright, Joseph Richardson, Joanna Empel, Julian Parkhill, Alex J. O’Neill

**Affiliations:** a School of Molecular and Cellular Biology, Faculty of Biological Sciences, University of Leeds, Leeds, United Kingdom; b Infection Biology Department, Faculty of Infectious and Tropical Diseases, London School of Hygiene & Tropical Medicine, London, United Kingdom; c Department of Epidemiology and Clinical Microbiology, National Medicines Institute, Warsaw, Poland; d Department of Veterinary Medicine, University of Cambridge, Cambridge, United Kingdom; McMaster University

**Keywords:** silenced antibiotic resistance, SARM, *Staphylococcus*

## Abstract

Sporadic literature reports describe isolates of pathogenic bacteria that harbor an antibiotic resistance determinant but remain susceptible to the corresponding antibiotic as a consequence of a genetic defect. Such strains represent a source from which antibiotic resistance may reemerge to cause treatment failure in patients. Here, we report a systematic investigation into the prevalence and nature of this phenomenon, which we term silencing of antibiotic resistance by mutation (SARM). Instances of SARM were detected among 1,470 Staphylococcus aureus isolates through side-by-side comparison of antibiotic resistance genotype (as determined by whole-genome sequencing) versus phenotype (as assessed through susceptibility testing). Of the isolates analyzed, 152 (10.3%) harbored a silenced resistance gene, including 46 (3.1%) that exhibited SARM to currently deployed antistaphylococcal drugs. SARM resulted from diverse mutational events but most commonly through frameshift mutation of resistance determinants as a result of point deletion in poly(A) tracts. The majority (∼90%) of SARM strains reverted to antibiotic resistance at frequencies of ≥10^−9^; thus, while appearing antibiotic sensitive in the clinical microbiology laboratory, most S. aureus isolates exhibiting SARM will revert to antibiotic resistance at frequencies achievable in patients. In view of its prevalence in a major pathogen, SARM represents a major potential threat to the therapeutic efficacy of antibiotics.

## Abstract (precis)

Antibiotic resistance hinders the treatment of bacterial infection. To guide effective therapy, clinical microbiology laboratories routinely perform susceptibility testing to determine the antibiotic sensitivity of an infecting pathogen. This approach relies on the assumption that it can reliably distinguish bacteria capable of expressing antibiotic resistance in patients, an idea challenged by the present study. We report that the important human pathogen Staphylococcus aureus frequently carries antibiotic resistance genes that have become inactivated (“silenced”) by mutation, leading strains to appear antibiotic sensitive. However, resistance can rapidly reemerge in most such cases, at frequencies readily achievable in infected patients. Silent antibiotic resistance is therefore prevalent, transient, and evades routine detection, rendering it a major potential threat to antibacterial chemotherapy.

## INTRODUCTION

Antibiotic resistance is dramatically undermining our ability to treat bacterial infection and constitutes a serious threat to public health (1). As the prevalence of antibiotic resistance increases among bacterial pathogens, so too does the importance of the role played by the clinical microbiology laboratory in determining which antibacterial drugs retain sufficient activity against a given isolate to offer potential therapeutic benefit. The gold standard approach for establishing this information is susceptibility testing, i.e., challenging the isolate with an antibacterial drug and assessing whether bacterial growth can be inhibited at concentrations likely to be achievable *in vivo*. This approach relies on the assumption that such testing will reliably distinguish bacteria capable of expressing antibiotic resistance in a patient. Unfortunately, this assumption does not always hold true.

Sporadic reports exist of bacteria harboring antibiotic resistance determinants that have become inactivated by a genetic defect (2–6), a phenomenon we term “silencing of antibiotic resistance by mutation” (SARM). By definition, strains subject to SARM ordinarily fail to express antibiotic resistance, and will therefore be classified as antibiotic sensitive upon susceptibility testing. However, SARM may in some cases be reversible (4, 5), thereby allowing the rapid reemergence of antibiotic resistance in an apparently antibiotic-susceptible strain. Where reversion of SARM to full phenotypic resistance occurs during treatment of infection with the corresponding antibacterial drug, this would prompt an unanticipated therapeutic failure. Indeed, a recent study described a case of such reversion to resistance occurring in a patient with the resultant failure of antibiotic treatment (6).

Information is currently lacking regarding the prevalence and scope of SARM in bacterial pathogens or the extent to which it is reversible; an appreciation of these aspects will be required to enable an assessment of the likely clinical impact of SARM on the effectiveness of antibacterial chemotherapy. We report here the first large, systematic screen to identify instances of SARM in an important bacterial pathogen of humans (Staphylococcus aureus). Our findings establish that SARM is prevalent, affects a broad range of antibiotic resistance determinants, and results from diverse genetic events that prevent expression of a functional resistance protein. Crucially, we demonstrate that SARM is in most cases readily reversible, revealing this phenomenon as an important potential source of therapeutic failure in the treatment of bacterial infection.

## RESULTS

### Prevalence of SARM in clinical isolates of S. aureus.

This study employed a collection of 1,470 S. aureus strains, most of which were recovered as the causative organism from human infections, and all of which are multidrug resistant (defined here as resistant to two or more clinically deployed antibacterial drug classes). The entire collection was subjected to susceptibility testing using a panel comprising the major antistaphylococcal drug classes in clinical use, and was in parallel subjected to whole-genome sequencing (WGS), *de novo* genome assembly, and *in silico* detection of acquired antibiotic resistance genes using the ARIBA tool. Staphylococci that appeared antibiotic sensitive according to published clinical breakpoints but harbored a cognate resistance gene were considered potential examples of SARM. However, some antibiotic resistance determinants (e.g., *blaZ*, *mecA*, and *ermA*) are known to require induction for resistance to manifest, and care was therefore taken to ensure that apparent instances of discordance between resistance genotype and phenotype did not simply constitute a failure to induce resistance. To assess this, all potential SARM isolates were preincubated with subinhibitory concentrations of the relevant antibiotic for 1 h before subjecting them to repeat susceptibility determinations.

**FIG 1:**
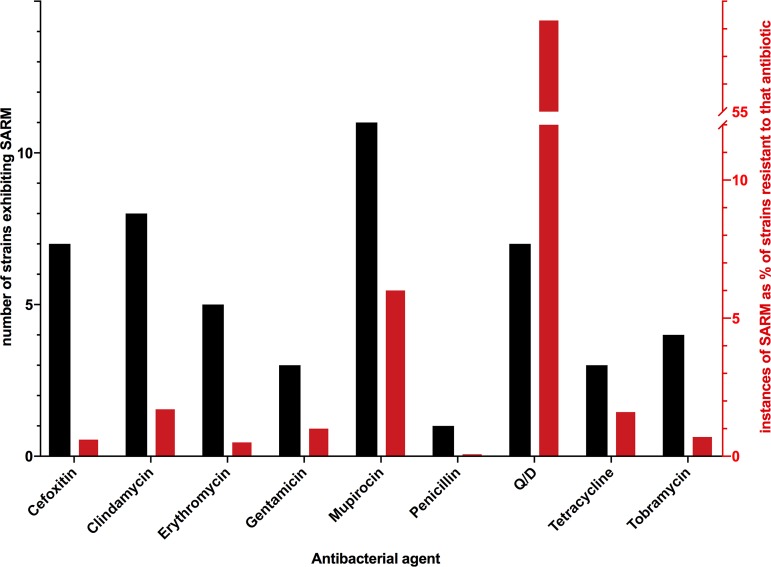
Prevalence of SARM to clinically deployed antibacterial drugs in S. aureus. The numbers of SARM strains identified for given agents are shown as black bars (left *y* axis), while the red bars (right *y* axis) show this value as a percentage of the total number of isolates exhibiting phenotypic resistance to that drug in this study. Two of the aminoglycoside SARM strains identified carry a silenced *aacA-aphD* gene that confers resistance to multiple aminoglycosides and have therefore been included in the total number of instances of SARM for both gentamicin and tobramycin.

Strains exhibiting SARM were identified for the aminoglycosides (involving the resistance genes *aacA-aphD* and *ant4*), β-lactams (cefoxitin [*mecA*] and penicillin [*blaZ*]), clindamycin [*vga*(*A*)*v*], erythromycin (*ermA*), mupirocin (*mupA*), quinupristin-dalfopristin (Q/D) [*vga*(*A*)*v* together with *ermA* or *ermC*], and tetracycline (*tetK* and *tetM*) (Table 1). In total, 46 (3.1%) of the isolates exhibited SARM to currently deployed antistaphylococcal drugs (Fig. 1), with one isolate (NRS752) harboring two silenced resistance genes (*mecA* and *blaZ*) (Table 1). We additionally identified numerous instances of SARM to spectinomycin, an antibiotic that is not used clinically to treat staphylococcal infection in humans but is employed for the treatment of gonorrhea and a variety of bacterial infections encountered in veterinary medicine (7, 8); ARIBA detected 21 strains with an apparently inactivated version of the spectinomycin resistance gene, *aad9*, which were confirmed in subsequent susceptibility tests to be spectinomycin sensitive (Table 1). ARIBA analysis also identified instances of mutationally inactivated resistance genes in strains exhibiting no discordance between genotype and phenotype, a situation attributable to the presence in those strains of a second, intact resistance gene that phenotypically masked the effect of SARM (see Table S1 in the supplemental material). Accordingly, we designated this phenomenon “masked SARM” (mSARM) to distinguish it from “true” SARM that impacts antibiotic resistance phenotype. For example, strain MOS330 harbored a *tetM* gene inactivated by a frameshift mutation, but it also carried an intact *tetK* gene that conferred resistance to tetracycline. Most strikingly, we identified 83 strains carrying apparently inactivated *blaZ* or *blaRI* genes but which were nonetheless resistant to penicillin owing to the fact that they also carried *mecA* (Table S1). Considering all examples of SARM and mSARM together, phenotypically silent antibiotic resistance genes were detected in 152 (10.3%) of the 1,470 strains analyzed.

**TABLE 1:** Nature of SARM strains identified in this study

Antibacterial drug class affected	Drug tested	Resistance gene(s)	No. of SARM strains identified	Representative strain	MIC (mg/liter)	Mutation	Consequence of mutation
Aminoglycosides/aminocyclitols	Gentamicin	*aacA-aphD*	3	GAL218	0.25	Insertion of transposable element IS*256* between nucleotides 834 and 835	Disruption of coding sequence
	Tobramycin	*aacA-aphD*	2	GAL218	0.5	Insertion of transposable element IS*256* between nucleotides 834 and 835	Disruption of coding sequence
		*ant4*	2	MOS258	0.25	Insertion of C between nucleotides 57 and 58	Frameshift
	Spectinomycin	*aad9*	4	NRS256	64	Single nucleotide deletion in poly(A) tract (189–195)	Frameshift
		*aad9*	1	MOS430	64	C_725_A nucleotide substitution (codon change TCA to TAA)	Nonsense mutation (S_242_Stop)
		*aad9*	16	MOS427	64	C_298_T nucleotide substitution (codon change CAA to TAA)	Nonsense mutation (Q_100_Stop)
β-Lactams	Cefoxitin	*mecA*	2	GAL206	4	Insertion of G between nucleotides 229 and 230	Frameshift
		*mecA*	4	SG138	4	Unknown	Unknown
		*mecA*	1	NRS752	4	Truncated *blaRI* regulator gene [single nucleotide deletion in poly(A) tract (466–473)]	Repression of *mecA* expression and no derepression in the presence of antibiotic
	Penicillin	*blaZ*	1	NRS752	0.03	Single nucleotide deletion in poly(A) tract (92–99)	Frameshift
Lincosamides	Clindamycin	*vga*(*A*)*v*	8	MOS244	0.25	Unknown	Unknown
Macrolides	Erythromycin	*ermA*	1	MOS55	0.5	Deletion in 5' leader (−1 to −48 and −62 to −144)	Loss of translation initiation elements^*a*^
		*ermA*	2	MOS283	0.5	Insertion of transposable element IS*256* between nucleotides 419 and 420	Disruption of coding sequence
		*ermA*	1	MOS287	0.5	C_574_T nucleotide substitution (codon change CGA to TGA)	Nonsense mutation (R_192_Stop)
		*ermA*	1	NRS720	0.5	G_112_A nucleotide substitution (codon change GGA to AGA)	Missense mutation G_38_R in binding pocket for cosubstrate
Streptogramins	Q/D	*vga*(*A*)*v/ermC*	6	MOS98	1	Unknown	Unknown
		*vga*(*A*)*v/ermA*	1	MOS119	1	Unknown	Unknown
Tetracyclines	Tetracycline	*tetK*	1	NRS699	0.5	Insertion of transposable element IS*431* upstream of coding region (−103 to −104)	Promoter separated from coding sequence
		*tetM*	2	MOS22	1	Single nucleotide deletion in poly(A) tract (777–784)	Frameshift
Miscellaneous	Mupirocin	*mupA*	11	DUB36	0.5	Single nucleotide deletion in poly(A) tract (272–280)	Frameshift

a Similar to a previous report of deletion of translation initiation elements of *ermA* (46).

10.1128/mBio.01755-19.2 TABLE S1 All S. aureus isolates identified in this study that contain a silenced antibiotic resistance gene (SARM and mSARM). Highlighted (alternating blue and green) are groups of isolates with identical SARM/mSARM genotypes that phylogenetic analysis indicates are clonally related. An asterisk in the ST (sequence type) column denotes a novel ST for which the closest match is given. CC, clonal complex. “Clustered” indicates whether isolates are grouped together with another SARM or mSARM isolate within the same monophyletic clade. Download Table S1, XLSX file, 0.02 MB. Copyright © 2019 Kime et al. 2019 Kime et al. This content is distributed under the terms of the Creative Commons Attribution 4.0 International license.

### Mutational events responsible for gene silencing in SARM strains.

**FIG 2:**
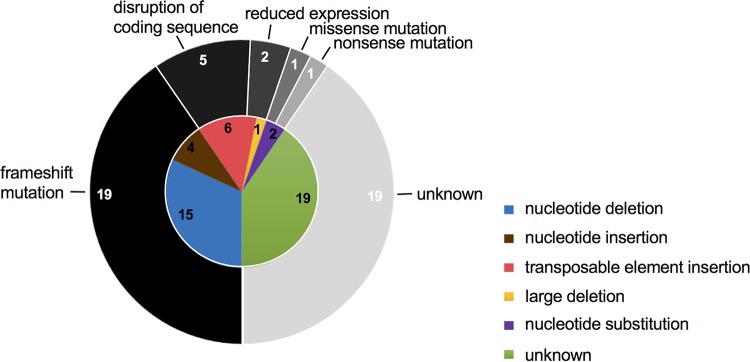
Genetic changes underlying SARM to clinically deployed antistaphylococcal drugs and the mechanism by which these changes lead to gene silencing. Classes of mutational event leading to antibiotic resistance gene silencing in SARM strains, and the number of examples detected in each case, are shown on the inner (colored) chart. The consequence of these changes for expression of the resistance gene is indicated on the outer (gray-scale) ring.

For approximately 70% (48 of 67) of SARM isolates, genetic changes were identified within either the coding region or the expression/regulatory elements of the resistance gene that could account for failure to produce a functional antibiotic resistance protein (Table 1). The genetic changes underlying SARM were diverse, running the gamut of common mutational events. In the majority of cases, SARM was the result of point mutations (insertions, deletions, and substitutions), though larger deletions and insertions involving integration of transposable elements were also identified (Fig. 2). The most common type of mutation identified was nucleotide deletion, which in all instances involved the loss of a single nucleotide from a poly(A) tract. Such mutations, which are thought to result from slippage of DNA polymerase during replication of these tracts (9), have previously been reported in isolated cases of SARM (6, 10). Collectively, the genetic changes identified resulted in loss of resistance gene function in a variety of ways, but frequently as a result of frameshift (Table 1 and Fig. 2).

### Reversion to phenotypic antibiotic resistance in strains exhibiting SARM.

To assess the potential for SARM strains to revert to phenotypic antibiotic resistance, we challenged representatives of all distinct SARM genotypes (and all strains in which the basis for SARM could not be defined) with the corresponding antibacterial agent at concentrations of ≥4× MIC. The majority (90%) of SARM strains yielded antibiotic-resistant colonies upon challenge, at frequencies between 10^−4^ and 10^−10^ (Table 2). Among strains for which a likely genetic basis for SARM had been defined, ∼96% showed reversion to phenotypic antibiotic resistance. Genetic characterization of revertants revealed that loss of SARM was often the result of direct reversion of the original silencing mutation (Table 2). The frequency of reversion of SARM resulting from shortening of poly(A) tracts was proportional to tract length, with longer tracts reverting more readily (see Fig. S1 in the supplemental material); this is as expected if reversion results from slipped-strand mispairing, since the opportunity for such events to occur will increase the longer the tract.

**TABLE 2:** Reversion to antibiotic resistance in SARM strains^*a*^

Antibacterial drug tested	Resistance gene(s)	Representative strain tested for reversion	Reversion frequency	Revertant MIC (mg/liter)/fold increase relative to SARM strain	Mechanism of reversion
Gentamicin	*aacA-aphD*	GAL218	2.9 (±1.1) × 10^−8^	32/128-fold	Excision of transposable element IS*256*
Tobramycin	*aacA-aphD*	GAL218	8.5 (±1.3) × 10^−7^	16/32-fold	Excision of transposable element IS*256*
	*ant4*	MOS258	8.1 (±2.7) × 10^−10^	64/256-fold	Deletion of nucleotide G_60_; amino acid change K_20_Q
Spectinomycin	*aad9*	NRS256	6.6 (±6.3) × 10^−9^	>512/>8-fold	Direct reversion of original mutation
	*aad9*	MOS430	1.8 (±0.7) × 10^−6^	>512/>8-fold	1. Direct reversion of original mutation
					2. T_724_C; amino acid S_242_Q
	*aad9*	MOS427	9.2 (±6.3) × 10^−7^	>512/>8-fold	1. Direct reversion of original mutation
					2. C_298_A; amino acid Q_100_K
Cefoxitin	*mecA*	GAL206	2.9 (±1.9) × 10^−9^	64/16-fold	1. Deletion of C_222_; amino acid V_75_L
					2. Deletion of A_228_; no coding change
					3. Deletion of A_232_; amino acid D_77_G and I_78_L
	*mecA*	SG138	1.1 (±0.2) × 10^−4^	32/8-fold	Unknown
	*mecA*	NRS752	4.0 (±2.1) × 10^−6^	32/8-fold	Loss of *bla* operon in 2 out of 3 revertants analyzed
Penicillin	*blaZ*	NRS752	1.4 (±1.3) × 10^−6^	8–16/256- to 512-fold	Unknown
Clindamycin	*vga*(*A*)*v*	MOS244	2.2 (±1.1) × 10^−9^	1/4-fold	Increased copy number of Tn*5406* [carries *vga*(*A*)*v*] from five to six
Erythromycin	*ermA*	MOS55	<4.8 (±1.7) × 10^−10^	None recovered	Not applicable
	*ermA*	MOS283	4.4 (±1.7) × 10^−7^	>256 (induced by erythromycin)/ >512-fold	Excision of transposable element IS*256*
	*ermA*	MOS287	1.9 (±1.3) × 10^−7^	>256 (induced by erythromycin)/ >512-fold	Direct reversion of original mutation
	*ermA*	NRS720	1.4 (±0.6) × 10^−9^	>256 (constitutive)/ >512-fold	Direct reversion of original mutation
Q/D	*vga*(*A*)*v/ermC*	MOS98	1.4 (±1.1) × 10^−9^	8/8-fold	Unknown
	*vga*(*A*)*v/ermA*	MOS119	<8.8~9 × 10^−10^	8/8-fold	Unknown
Tetracycline	*tetK*	NRS699	5.1 (±1.8) × 10^−6^	64/128-fold	Unknown
	*tetM*	MOS22	7.9 (±3.3) × 10^−8^	128/128-fold	Direct reversion of original mutation
Mupirocin	*mupA*	DUB36	1.4 (±1.1) × 10^−7^	>256/>512-fold	Direct reversion of original mutation

a Reversion studies were conducted using a single strain corresponding to each distinct SARM genotype, and reversion frequencies are expressed as means ± standard deviations. For MOS119, revertants were not recovered in all determinations, and consequently only an approximate reversion frequency is given.

10.1128/mBio.01755-19.1 FIG S1 Frequency of reversion at homopolymeric tracts in SARM strains as a function of tract length. Values are means ± 1 standard deviation (error bars). Download FIG S1, PDF file, 0.10 MB. Copyright © 2019 Kime et al. 2019 Kime et al. This content is distributed under the terms of the Creative Commons Attribution 4.0 International license.

### Representative examples of mutational events underlying reversion of SARM.

**FIG 3:**
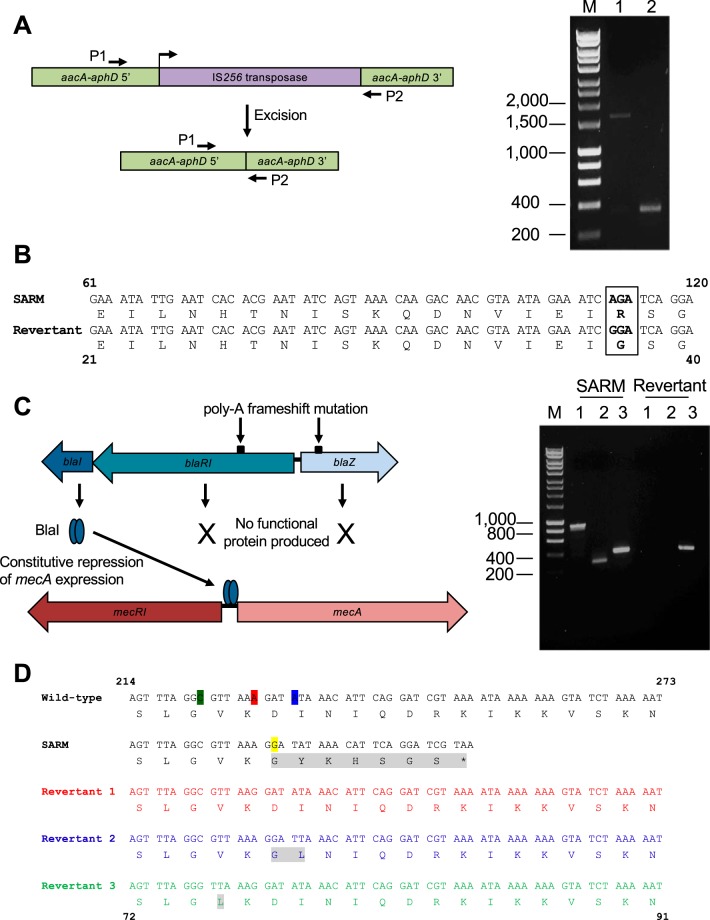
Representative examples of genetic events leading to reversion of SARM in S. aureus. (A) The diagram (left) shows the basis for SARM in S. aureus GAL218 (refer to text for details). (Right) PCR amplification with oligonucleotide primers P1 and P2 generated a PCR product of ∼1.7 kb from this strain (lane 1). Reversion to phenotypic resistance was associated with excision of this transposable element, as revealed by the generation of a smaller PCR amplicon (∼370 bp) from the revertant (lane 2). The lane labeled M contains 1-kb Hyperladder (Bioline). (B) Reversion of SARM in strain NRS720. The genetic defect in the *ermA* gene is shown boxed, with nucleotide numbers relative to the translation start codon indicated above the sequences and amino acid numbers shown below the sequences. (C) The diagram (left) shows the basis for SARM in strain NRS752 (see the text for details). (Right) The *blaZ-blaRI* (lanes 1; ∼950-bp) and *blaI* (lanes 2; ∼300-bp) genes could be amplified by PCR from the SARM strain but were absent in a cefoxitin-resistant revertant. Amplification of the *spa* gene (lanes 3; ∼450 bp) provided a PCR control. (D) Basis for reversion of SARM in strain GAL206. The nucleotide and amino acid sequences are labeled as in panel B, with amino acids that differ from those in the wild type shown in gray-scale. The insertion inactivating *mecA* in this strain is highlighted in yellow, and the nucleotide that has become deleted in each revertant to restore phenotypic resistance is indicated in the wild-type sequence in the corresponding color.

SARM strain GAL218 harbors the *aacA-aphD* gene that encodes the bifunctional aminoglycoside modifying enzyme AAC(6′)-APH(2′′) and that ordinarily confers resistance to both gentamicin and tobramycin. DNA sequence analysis of this strain identified the presence of an IS*256* transposable element inserted between nucleotides 834 and 835 of this gene, which thereby prevented expression of an intact resistance protein (Fig. 3A). Reversion to gentamicin and tobramycin resistance occurred at frequencies of 10^−7^ to 10^−8^ as a result of spontaneous excision of IS*256* to restore the integrity of the gene (Table 2 and Fig. 3A).

SARM can result from a single missense mutation in the coding region of an antibiotic resistance determinant. Such a genetic defect was found in strain NRS720 within *ermA*, a gene encoding a methyltransferase that modifies rRNA to mediate erythromycin resistance. Nucleotide substitution G_112_A produced amino acid change G_38_R within the ErmA binding pocket for the cosubstrate, *S*-adenosyl-l-methionine (SAM), with resultant loss through substitution of a residue essential for enzyme activity (Fig. 3B) (11). Revertants exhibiting high-level erythromycin resistance were selected upon challenge with the antibiotic at frequencies of ∼10^−9^, and sequence analysis of the *ermA* gene confirmed direct reversion of the original mutation to reinstate G_38_ (Table 2 and Fig. 3B).

In the absence of functional MecRI-MecI regulatory proteins, expression of the methicillin resistance gene *mecA* can be regulated by the homologous BlaRI-BlaI system (12, 13). In SARM strain NRS752, the MecI transcriptional repressor is absent, and expression of *mecA* is subject to repression by BlaI. However, the sensor/signal transducer gene (*blaRI*) in that strain contains a single nucleotide deletion within a poly(A) tract that results in a frameshift mutation (Table 1 and Fig. 3C); sensing of antibiotic and consequent proteolysis of BlaI is therefore impaired, and *mecA* expression is subject to continual repression. Cefoxitin-resistant revertants were selected at high frequency (10^−6^) upon challenge with antibiotic and resulted from complete loss of the *blaZ-blaRI-blaI* operon in two of three revertants analyzed (Table 2 and Fig. 3C).

Reversion to antibiotic resistance in strains exhibiting SARM can also occur through suppression, an indirect mechanism in which the original silencing mutation remains, but compensatory mutation allows expression of a functional resistance protein. A guanine nucleotide inserted into *mecA* between positions 229 and 230 was responsible for inactivating this gene by frameshift mutation in SARM strain GAL206 (Table 1). Analysis of three cefoxitin-resistant revertants recovered following selection with the antibiotic revealed each to have undergone deletion of a different nucleotide in the vicinity of the original insertion site (C_222_, A_228_, and A_232_) to restore the reading frame and allow production of a functional resistance protein (Table 2 and Fig. 3D). These mutations resulted in minor coding changes in two of the three revertants relative to the wild-type sequence (V_75_L and D_77_G/I_78_L), but these changes did not appear to impair the ability of the protein to mediate cefoxitin resistance.

### Clonality and context of isolates harboring silenced antibiotic resistance genes.

Existing, publicly available collections of S. aureus genomes were compiled to contextualize isolates analyzed in this study and included collections with a high diversity of S. aureus clonal complexes (14–17) and those comprising isolates from the same clonal complex but broad geographical coverage (18–23). Subsequent construction of RAxML phylogenies (24) allowed determination of the clonality of SARM/mSARM isolates and delineated their relationship to S. aureus strains from European and global collections.

**FIG 4:**
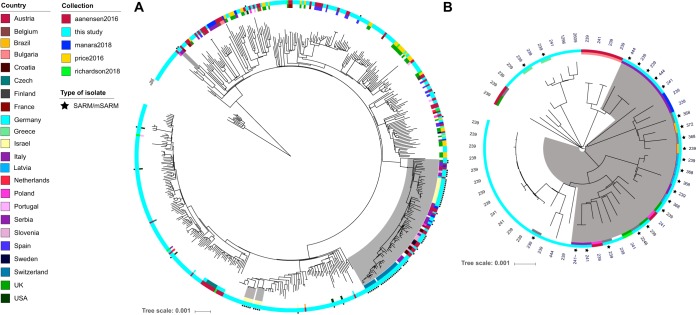
Core genome phylogenies of S. aureus isolates from this and contextual studies. (A) All clonal complex 5 isolates. (B) The ST239 clade of clonal complex 8. In each case, the outer ring indicates the collection, while the inner ring shows the country of origin of isolates from contextual collections and isolates from this study that harbor a silenced antibiotic resistance determinant. SARM/mSARM isolates from this study are denoted with a star, with clustered isolates shaded in gray.

Of the 67 SARM isolates, 39 (~58%) clustered with at least one other isolate with an identical SARM genotype, implying clonality (Table S1). This value rose to ~72% (110/152) when considering all isolates (both SARM and mSARM) that harbor a silenced antibiotic resistance gene (Table S1). Contextual isolates revealed that two clades carrying silenced antibiotic resistance genes are widely spread geographically; a large monophyletic clade made up of mostly ST228 and ST111 isolates (clonal complex 5 [CC5]) spanning Europe and Israel (*n *= 56 SARM isolates) (Fig. 4A) and a large CC8 clade made up of mostly ST239 isolates (*n *= 23) (Fig. 4B), spanning multiple European countries. In view of the fact that the CC5 clone exhibits mSARM and the CC8 clade is predominantly associated with SARM to an antibiotic not used to treat staphylococcal infection in humans (spectinomycin), resistance gene silencing in these clades does not for the most part have obvious clinical relevance. The other clades identified were smaller (2 to 11 isolates) and restricted to the same country (Table S1), demonstrating that SARM/mSARM occur across a diverse background of S. aureus clades.

## DISCUSSION

We report here that silenced antibiotic resistance genes are prevalent in one of the major bacterial pathogens of humans, occurring in ∼10% of S. aureus isolates tested, including 3.1% that exhibit SARM to currently deployed antistaphylococcal agents. Carriage and expression of antibiotic resistance genes are known to exact a fitness cost on bacteria in antibiotic-free environments (25), and the phenomenon of SARM can be understood in terms of counterselection of these costly determinants when not required. Key to assessing the potential medical impact of these findings is whether this loss of resistance occurs primarily in the clinic or is instead an event that takes place during passage or storage of these strains in the laboratory after isolation. The independent recovery of closely related S. aureus isolates with identical SARM genotypes strongly supports the idea that SARM preexisted laboratory isolation in these cases, and thereby implies that—at minimum—58% of SARM strains identified in this study arose in the clinical environment (Table S1).

The genetic basis for SARM could be defined in the majority of isolates analyzed, usually involving mutations lying within—or upstream of—the antibiotic resistance determinant that prevented expression of a functional resistance protein. However, the basis for SARM remains undefined in a minority of isolates that harbor intact antibiotic resistance genes associated with appropriate/undamaged upstream expression signals (Table 1). In a proportion of the latter isolates, it could be that as-yet-unidentified *trans*-acting elements are responsible for silencing resistance; in line with this idea, such elements have previously been implicated in silencing of plasmid-borne antibiotic resistance genes in Escherichia coli strains recovered from animal experiments (26). In most cases, however, a more prosaic explanation exists. Our study identified several strains carrying an apparently wild-type (i.e., fully functional) version of the *vga*(*A*)*v* gene but which nonetheless exhibited susceptibility to clindamycin. WGS analysis of a clindamycin-resistant variant recovered from one of these strains upon selection detected an increase from five to six in the copy number of Tn*5406* [the transposable element that carries *vga*(*A*)*v*] (Table 2), a change that appears to cause resistance by increasing the gene dosage of *vga*(*A*)*v*. While this finding does not exclude the possibility that this genetic change is acting to overcome a silencing mutation/element lying distant from the resistance gene, we consider it more likely that expression of *vga*(*A*)*v* is ordinarily simply insufficient to confer clinically significant resistance to clindamycin in these strains (i.e., resistance gene expression is suboptimal rather than silenced by mutation) (27) and that resistant variants recovered upon antibiotic challenge are gain-of-function mutants rather than revertants. Suboptimal—rather than silenced—resistance gene expression would also appear a more plausible explanation for the observation that 58% (7 of 12) of strains found to carry the genes for resistance to Q/D were nonetheless susceptible to the drug, a value an order of magnitude greater than that for SARM cases detected for the other antibacterial drug classes tested (Fig. 1). If lack of an antibiotic resistance phenotype in some of these strains is indeed the consequence of suboptimal resistance gene expression, then they would not—by definition—qualify under the heading of SARM. Nevertheless, the implications for potential therapeutic failure are ultimately the same whether phenotypic antibiotic resistance becomes reinstated in such strains through reversion of true SARM or occurs for the first time through *de novo* activation of a previously cryptic resistance genotype.

SARM can be irreversible, involving a permanent loss of antibiotic resistance, or reversible; the latter implies that phenotypic resistance may be regained and that silencing of resistance in such cases must therefore be considered effectively transient. In the overwhelming majority of strains, SARM was found to be reversible, yielding progeny exhibiting phenotypic antibiotic resistance upon challenge with the corresponding antibiotic. SARM reversion occurred at frequencies (generally ≥10^−9^) readily attainable within bacterial populations during infection (28). Indeed, antistaphylococcal drugs that select resistance at similar frequencies (e.g., rifampin, fusidic acid) are rarely used as monotherapy for serious infections precisely to avoid therapeutic failure resulting from resistance development during the treatment interval (29). Furthermore, as indicated above, reversion to antibiotic resistance in a SARM isolate has been documented in a patient during treatment (6). Thus, most of the SARM strains identified in this study would be anticipated to revert to full phenotypic antibiotic resistance in patients upon initiation of therapy with the corresponding antibacterial drug, with consequent therapeutic failure.

The finding that SARM is both prevalent and frequently reversible in a major bacterial pathogen challenges a key assumption of *in vitro* susceptibility testing: that it represents a reliable predictor of the microbiological response to an antibacterial drug *in vivo*. Since susceptibility testing cannot discriminate truly antibiotic-sensitive isolates from those exhibiting SARM, it will in a proportion of cases support the clinical deployment of an antibacterial drug to treat infection for which therapeutic failure lies only a reversion event away. In effect, SARM represents a class of “hidden” antibiotic resistance that evades routine detection, and therefore has parallels with the recently dissected phenomenon of heteroresistance (30). There is no easy fix for this issue at present, given that the methods by which SARM can be detected are not generally feasible in the context of the clinical microbiology laboratory. However, growing interest in—and proof of principle for—the use of WGS to predict antibiotic resistance *in silico* suggests that DNA sequencing could ultimately supplant routine susceptibility testing, a development that would address the problem (14, 31); detection of antibiotic resistance by genotype irrespective of phenotype has its own limitations and pitfalls, but it does have the benefit of identifying potential SARM strains. Until such time, we recommend that, wherever practicable, SARM be investigated as a potential cause in cases of unanticipated therapeutic failure in the treatment of bacterial infection.

## MATERIALS AND METHODS

### Bacteria used in this study.

To ensure maximal diversity, S. aureus isolates were sourced from a broad range of clinical and geographical settings. We employed three major preexisting collections of S. aureus; an established resource comprising diverse isolates held at the University of Leeds (*n = *320) (32), a subset of isolates from the NARSA collection (*n = *241; https://www.beiresources.org/), and a set of 496 methicillin-resistant S. aureus (MRSA) strains collected by the MOSAR consortium from sites across Europe and Israel (33–35). Further isolates were obtained from several United Kingdom sites that included St. Georges NHS Trust, London (*n = *157), the Royal Veterinary College, Hertfordshire (*n = *87), and hospitals in the Republic of Ireland (*n = *169). The majority of strains were isolated from human patients (*n *= 1437), with a small number deriving from animal sources (*n *= 33). The laboratory strain S. aureus SH1000 was used as an antibiotic-susceptible control organism (36).

### Media, antibiotics, and susceptibility testing.

MICs were determined by agar dilution according to CLSI guidelines (37), using cation-adjusted Mueller-Hinton agar (MHA) (Becton, Dickinson) and broth (MHB) (Sigma-Aldrich). To facilitate susceptibility testing of the entire strain collection, only a small range of antibacterial drug concentrations around the EUCAST clinical breakpoints were employed to distinguish sensitive and resistant isolates in the first instance (38). Susceptibility determinations were performed with the following antistaphylococcal drugs: aminoglycosides (gentamicin and tobramycin), glycopeptides (teicoplanin and vancomycin), β-lactams (cefoxitin and penicillin), macrolides/lincosamides/streptogramins (clindamycin, erythromycin, and quinupristin-dalfopristin [Q/D]), oxazolidinones (linezolid), tetracycline, and miscellaneous agents (chloramphenicol, fosfomycin, fusidic acid, mupirocin, and trimethoprim). In addition, susceptibility determinations with spectinomycin were performed on selected strains. All drugs were obtained from Sigma-Aldrich, with the exception of linezolid, tobramycin, and vancomycin (Cayman Chemical), penicillin (Fisher Scientific), and Q/D (Santa Cruz Biotechnology).

### Whole-genome sequencing and detection of resistance determinants.

Whole-genome sequencing (WGS) of S. aureus isolates was performed at the Wellcome Sanger Institute, UK. DNA libraries (450-bp insert size) were generated and sequenced using 100-bp paired-end reads on the Illumina HiSeq2000 platform, essentially as described previously (39). *De novo* genome assembly was performed as previously described (40), and sequence data were deposited with the European Nucleotide Archive (www.ebi.ac.uk/ena; accession numbers listed in Table S2 in the supplemental material). Assembled genomes were interrogated for acquired resistance genes using the Antimicrobial Resistance Identification By Assembly (ARIBA) tool (41), employing a custom-made database (Table S3) comprising only horizontally acquired genes documented to confer antibiotic resistance in staphylococci. The genetic basis for SARM was explored through analysis of genome sequence data and confirmed by PCR amplification (using Phusion DNA polymerase [New England Biolabs] according to the manufacturers’ instructions and oligonucleotide primers listed in Table S4), followed by DNA sequencing.

10.1128/mBio.01755-19.3 TABLE S2 Whole-genome accession numbers for strains sequenced in this study. Download Table S2, XLSX file, 0.1 MB. Copyright © 2019 Kime et al. 2019 Kime et al. This content is distributed under the terms of the Creative Commons Attribution 4.0 International license.

10.1128/mBio.01755-19.4 TABLE S3 Custom database of horizontally acquired genes used for ARIBA-based interrogation of whole-genome sequences. Download Table S3, DOCX file, 0.01 MB. Copyright © 2019 Kime et al. 2019 Kime et al. This content is distributed under the terms of the Creative Commons Attribution 4.0 International license.

10.1128/mBio.01755-19.5 TABLE S4 Oligonucleotide primers used in this study. Download Table S4, DOCX file, 0.01 MB. Copyright © 2019 Kime et al. 2019 Kime et al. This content is distributed under the terms of the Creative Commons Attribution 4.0 International license.

### Genomic and phylogenic analyses.

Publicly available collections of S. aureus genomes were compiled to contextualize isolates analyzed in this study. These collections included a pan-European structured survey of invasive S. aureus isolates (*n *= 308) (14) and a global collection of strains that represents the breadth of known clonal, geographical, and host species diversity of S. aureus (*n *= 800) (15), from which only human-infecting and colonizing isolates were selected (*n *= 437 of 800). In addition to the European and global collections, isolates were included from two large hospital-based studies from the United Kingdom (*n *= 1,977) (17) and Italy (*n *= 184) (16) that capture the most common S. aureus clonal complexes in European hospitals. These hospital studies were deduplicated to keep only one isolate per patient (the earliest available one) and one isolate per outbreak clone (the earliest observed) by removing consecutive and genetically related isolates (defined as having less than 50 single nucleotide polymorphisms [SNPs] at the core genome) from different patients. We ultimately retained 491 (of 1,977) and 110 (of 184) isolates from the United Kingdom and Italian hospital studies, respectively. In addition to including collections with a diversity of clonal complexes (CCs) (14–17), collections of isolates were compiled from the same clonal complex but broad geographical coverage for clonal complex 22 (CC22) (19), CC8 (18, 22), and CC398 (20, 23).

Short reads of all S. aureus genomes from this study and contextual collections were subjected to *de novo* assembly using Velvet (42) or Spades (43), and raw assemblies were improved using an established bacterial assembly and improvement pipeline (40). Sequence types (STs) were derived from *de novo* assemblies by extracting all seven S. aureus multilocus sequence type (MLST) loci and comparing them to the PubMLST database (www.PubMLST.org). Clonal complexes were derived from the allelic profile, allowing up to two allele mismatches from the reference ST. All isolates were mapped using SMALT v0.7.4 to the EMRSA15 ST22 reference genome (strain HO 5096 0412, GenBank accession number HE681097). SNPs at the chromosomal regions corresponding to the core genome were kept from whole-genome alignments, and maximum likelihood trees were created using RAxML (24) for each CC as previously described (44). The S. aureus species core genome was derived running Roary (45) on the global contextual collection with default settings (44), in this case using all 800 S. aureus isolates, which consisted of 1,766 genes and covered 1.76 Mb (62%) of the EMRSA15 ST22 reference genome.

### Selection and characterization of SARM revertants.

Selection of antibiotic-resistant revertants from strains exhibiting SARM was achieved by plating cultures onto MHA containing 4× to 128× MIC of the corresponding antibacterial agent and incubating for 48 h at 37°C. To calculate reversion frequencies, three independent cultures were sampled in triplicate to determine both revertant count and total count (determined by plating onto drug-free MHA), and results were expressed as the mean value for the number of revertants obtained/number of viable cells. Genetic changes were detected in SARM revertants by PCR amplification, followed by either DNA sequencing or sizing of PCR amplicons by agarose gel electrophoresis.

### Data availability.

All sequence data have been deposited with the European Nucleotide Archive and accession numbers are listed in Table S2.
