# Supplementary material for: Transient Silencing of Antibiotic Resistance by Mutation Represents a Significant Potential Source of Unanticipated Therapeutic Failure
Source: mBio. 2019 Oct 29;10(5):e01755-19. doi: 10.1128/mBio.01755-19 (PMC6819657; doi:10.1128/mBio.01755-19)

Figure S1. Frequency of reversion at homopolymeric tracts in SARM strains as a function of tract length. Values are means ± 1 standard deviation (error bars).

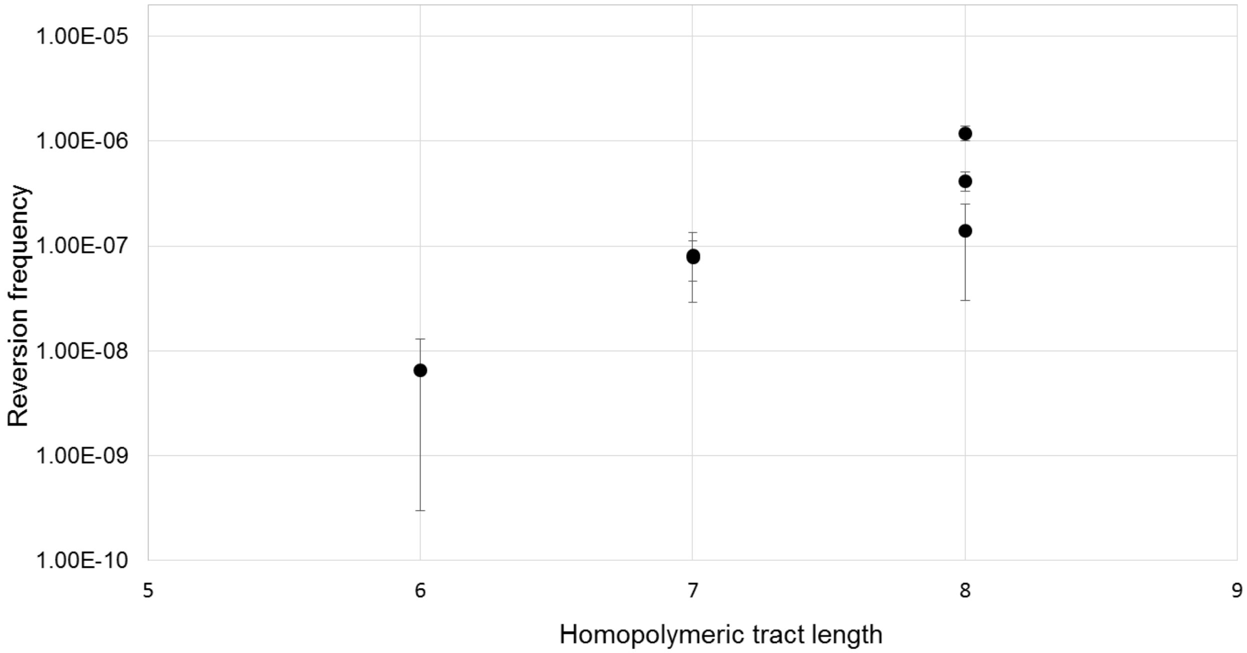

Supplement: FIG S1 [file mBio.01755-19-sf001.pdf]
