# Supplementary material for: Transient Silencing of Antibiotic Resistance by Mutation Represents a Significant Potential Source of Unanticipated Therapeutic Failure
Source: mBio. 2019 Oct 29;10(5):e01755-19. doi: 10.1128/mBio.01755-19 (PMC6819657; doi:10.1128/mBio.01755-19)
Supplement: TABLE S3 [file mBio.01755-19-st003.docx]

**Table S3.** Custom database of horizontally acquired genes used for ARIBA-based interrogation of whole-genome sequences.

| **Resistance gene** | **Accession number** | **Representative antibacterial drugs to which gene confers resistance** |
| --- | --- | --- |
| *ermA* | NC_009632 | erythromycin, clindamycin, quinupristin |
| *ermB* | CP026081 | erythromycin, clindamycin, quinupristin |
| *ermC* | NG_047809 | erythromycin, clindamycin, quinupristin |
| *erm33* | NC_005076 | erythromycin, clindamycin, quinupristin |
| *ermT* | FN390947 | erythromycin, clindamycin, quinupristin |
| *ermY* | AB014481 | erythromycin, clindamycin, quinupristin |
| *mefA* | AY064721 | erythromycin |
| *msrA* | CP012118 | erythromycin, quinupristin |
| *msrB* | M81802 | erythromycin, quinupristin |
| *msrD* | NG_048005 | erythromycin, quinupristin |
| *mphC* | NG_047989 | erythromycin |
| *ereA* | NG_047765 | erythromycin |
| *ereB* | A15097 | erythromycin |
| *lnuA* | NG_047914 | clindamycin |
| *lnuB* | CP031839 | clindamycin |
| *lsaB* | CP028164 | clindamycin, dalfopristin |
| *lsaE* | JX560992 | clindamycin, dalfopristin |
| *salA* | KC693025 | clindamycin, dalfopristin |
| *vga(A)* | NG_048552 | clindamycin, dalfopristin |
| *vga(A)_LC_* | DQ823382 | clindamycin, dalfopristin |
| *vga(A)v* | AF186237 | clindamycin, dalfopristin |
| *vgaB* | U82085 | clindamycin, dalfopristin |
| *vgaC* | FN377602 | clindamycin, dalfopristin |
| *vgaE* | FR772051 | clindamycin, dalfopristin |
| *vgbA* | M20129 | quinupristin |
| *vgbB* | AF015628 | quinupristin |
| *vatA* | L07778 | dalfopristin |
| *vatB* | U19459 | dalfopristin |
| *vatC* | AF015628 | dalfopristin |
| *cfr* | AM408573 | clindamycin, dalfopristin, linezolid, chloramphenicol |
| *tetK* | NC_013452 | tetracycline |
| *tetM* | CP035415 | tetracycline |
| *tetL* | FN377602 | tetracycline |
| *tetS* | KX774481 | tetracycline |
| *optrA* | MF805731 | linezolid, chloramphenicol |
| *fusB* | AY047358 | fusidic acid |
| *fusC* | NC_002953 | fusidic acid |
| *fusD* | CP022093 | fusidic acid |
| *dfrA* | GU565967 | trimethoprim |
| *dfrK* | NG_047759 | trimethoprim |
| *dfrG* | AB205645 | trimethoprim |
| *mupA* | X75439 | mupirocin |
| *mupB* | JQ231224 | mupirocin |
| *catA2* | CP020741 | chloramphenicol |
| *catA1* | P00486 | chloramphenicol |
| *catA3* | P00485 | chloramphenicol |
| *fexA* | AJ549214 | chloramphenicol |
| *cmlA* | LC093094 | chloramphenicol |
| *blaZ* | CP030136 | penicillin |
| *blaRI/blaI* | AB699881 | penicillin |
| *blaZ_LGA251* | FR821779 | penicillin |
| *mecA* | KC243783 | penicillin, cefoxitin |
| *mecRI* | NC_009487 | penicillin, cefoxitin |
| *mecI* | BA000018.3 | penicillin, cefoxitin |
| *mecA1* | Y13094 | penicillin, cefoxitin |
| *mecB* | NC_011996 | penicillin, cefoxitin |
| *mecC* | FR821779 | penicillin, cefoxitin |
| *vanA* | AE017171 | vancomycin, teicoplanin |
| *vanZ* | AE017171 | teicoplanin |
| *aad9* | CP030138 | spectinomycin |
| *ant(4')-Ia* | CP030136 | tobramycin |
| *aacA-aphD* | NC_005024 | gentamicin, tobramycin |
| *fosB* | CP012757 | fosfomycin |
| *fosB4* | NG_054661 | fosfomycin |
