# Supplementary material for: Transient Silencing of Antibiotic Resistance by Mutation Represents a Significant Potential Source of Unanticipated Therapeutic Failure
Source: mBio. 2019 Oct 29;10(5):e01755-19. doi: 10.1128/mBio.01755-19 (PMC6819657; doi:10.1128/mBio.01755-19)
Supplement: TABLE S4 [file mBio.01755-19-st004.docx]

**Table S4.** Oligonucleotide primers used in this study.

| **Gene** | **Primer sequence (5' to 3')** |
| --- | --- |
| *aacA-aphD* | GCCACAAATGTTAAGGCAATGA |
|  | ACTGGCAATATCTCGTTTTAACAA |
| *aad9* | GGTGGTTTACGCATTAACAGC |
|  | ACTTAACGAGTGCTTTCACCT |
| *ant4* | TGGACCAATAATAATGACTAGAGAAGA |
|  | GCACAAATCGCATCGTGGAA |
| *blaI* | TGGTTATTTTCTGGACACTCTCA |
|  | CCGTCTGGGAAAAATGCTGA |
| *blaZ-blaRI* | AAAGTATCTCTATTTTTAGCATGAGCA |
|  | TCGCTTTTGAAGTCGAAGCA |
| *ermA* | AGCGGTAAACCCCTCTGAGA |
|  | GCTTCAAAGCCTGTCGGAAT |
|  | CCAGAAAAACCCTAAAGACACGC |
|  | GTCACTTGACATAAGCCTCCA |
| *mecA* | AGTTGTAGTTGTCGGGTTTGGT |
|  | TGCTGTTCCTGTATTGGCCA |
|  | TGGAGACGAGCACTAATAACCA |
|  | CGTTCAGTCATTTCTACTTCACCA |
| *mupA* | AACTGCAAATGGCCTTCCT |
|  | TAGGGGGAGTCCATGTCAAC |
| *spa* | TAAAGACGATCCTTCGGTGAGC |
|  | CAGCAGTAGTGCCGTTTGCTT |
| *tetK* | CGCACGAATATTGAACCGACA |
|  | CAAAGCCAAAATAAAACGCTAT |
| *tetM* | GCATTGGAACTCGAACAAGAG |
|  | GTAGAGGGTGCGGATTTTCA |
| *vga(A)v* | AGCGAGGATAAAAGAATTAGAGGA |
|  | ACCACACTCCTTCAACCTCA |
